# Impact of the papillary muscles on cardiac magnetic resonance image analysis of important left ventricular parameters in hypertrophic cardiomyopathy

**DOI:** 10.1007/s12471-016-0805-y

**Published:** 2016-02-25

**Authors:** D.H.F. Gommans, J. Bakker, G.E. Cramer, F.W.A. Verheugt, M.A. Brouwer, M.J.M. Kofflard

**Affiliations:** Department of Cardiology, Radboud University Medical Centre, Nijmegen, The Netherlands; Department of Radiology, Albert Schweitzer Hospital, Dordrecht, The Netherlands; Department of Cardiology, Albert Schweitzer Hospital, Dordrecht, The Netherlands

**Keywords:** Hypertrophic cardiomyopathy, Papillary muscles, Cardiac magnetic resonance, CMR

## Abstract

**Purpose:**

The use of cardiac magnetic resonance (CMR) analysis has increased in patients with hypertrophic cardiomyopathy (HCM). Quantification of left ventricular (LV) measures will be affected by the inclusion or exclusion of the papillary muscles as part of the LV mass, but the magnitude of effect and potential consequences are unknown.

**Methods:**

We performed Cine-CMR in (1) clinical HCM patients (*n* = 55) and (2) subclinical HCM mutation carriers without hypertrophy (*n* = 14). Absolute and relative differences in LV ejection fraction (EF) and mass were assessed between algorithms with and without inclusion of the papillary muscles.

**Results:**

Papillary muscle mass in group 1 was 6.6 ± 2.5 g/m^2^ and inclusion of the papillary muscles resulted in significant relative increases in LVEF of 4.5 ± 1.8 % and in LV mass of 8.7 ± 2.6 %. For group 2 these figures were 4.0 ± 0.9 g/m^2^, 3.8 ± 1.0 % and 9.5 ± 1.8 %, respectively. With a coefficient of variation of 4 %, this 9 % difference in LV mass during CMR follow-up will be considered a change, while in fact the exact same mass may have been assessed according to two different algorithms.

**Conclusions:**

In clinical HCM patients, CMR quantification of important LV measures is significantly affected by inclusion or exclusion of the papillary muscles. In relative terms, the difference was similar in subjects without hypertrophy. This underscores a general need for a uniform approach in CMR image analysis.

## Introduction

Over the past few years, the use of cardiac magnetic resonance (CMR) imaging has steadily increased in patients with hypertrophic cardiomyopathy (HCM). Although CMR is not yet part of the current risk stratification [1], several studies have been undertaken to address the impact of CMR for future patient management [2, 3]. In recent publications it was demonstrated that left ventricular (LV) ejection fraction (EF) and LV mass can be used to identify HCM patients at high risk of adverse cardiac events [2, 4–6]. CMR is the current gold standard for quantification of these parameters, because of its superior accuracy and reproducibility [7].

In daily practice, uniformity in CMR image analysis is lacking on how to deal with measurements of the papillary muscles, despite recommendations in the current CMR guidelines. The guidelines state that LV volumes and LV mass should be quantified according to the same protocol as used for the reference ranges [8, 9]. In general, studies on normal values of LV parameters used to include the papillary muscles in the LV mass [10–12]. Surprisingly, in most general hospitals exclusion of the papillary muscles has become the standard [13].

In HCM patients the papillary muscle mass is higher than in normal healthy volunteers [14]. Therefore, especially in the HCM population, the impact of the papillary muscles on quantification of LV parameters might be substantial. We studied the impact of inclusion and exclusion of the papillary muscles on the quantification of LVEF and LV mass in clinical HCM patients with overt hypertrophy. In addition, we studied the impact in a group of subclinical HCM mutation carriers without hypertrophy.

## Materials and methods

### Study population

Study participants were recruited from a population that visited a specialised HCM outpatient clinic, which routinely performs repeated echocardiographic imaging, clinical follow-up and genetic testing according to a cascade strategy. Between April 2008 and May 2011 we systematically asked them to participate in a study program, which also included CMR analysis [15]. Patients with a history of septal reduction therapy, with LV hypertrophy due to other disorders that explain the myocardial hypertrophy (amyloidosis, MELAS, Anderson-Fabry etc.) or any contraindication to CMR imaging were not eligible.

Participants in the present study were either patients with an echocardiographically proven HCM according to the ACC/ESC guidelines [16] or subjects with an HCM-related pathogenic mutation without hypertrophy on echocardiography. The former will be referred to as clinical HCM patients (group 1, with overt hypertrophy), the latter will be referred to as subclinical HCM mutation carriers (group 2, without hypertrophy).

### CMR image analysis: handling of papillary muscles

**Fig. 1:**
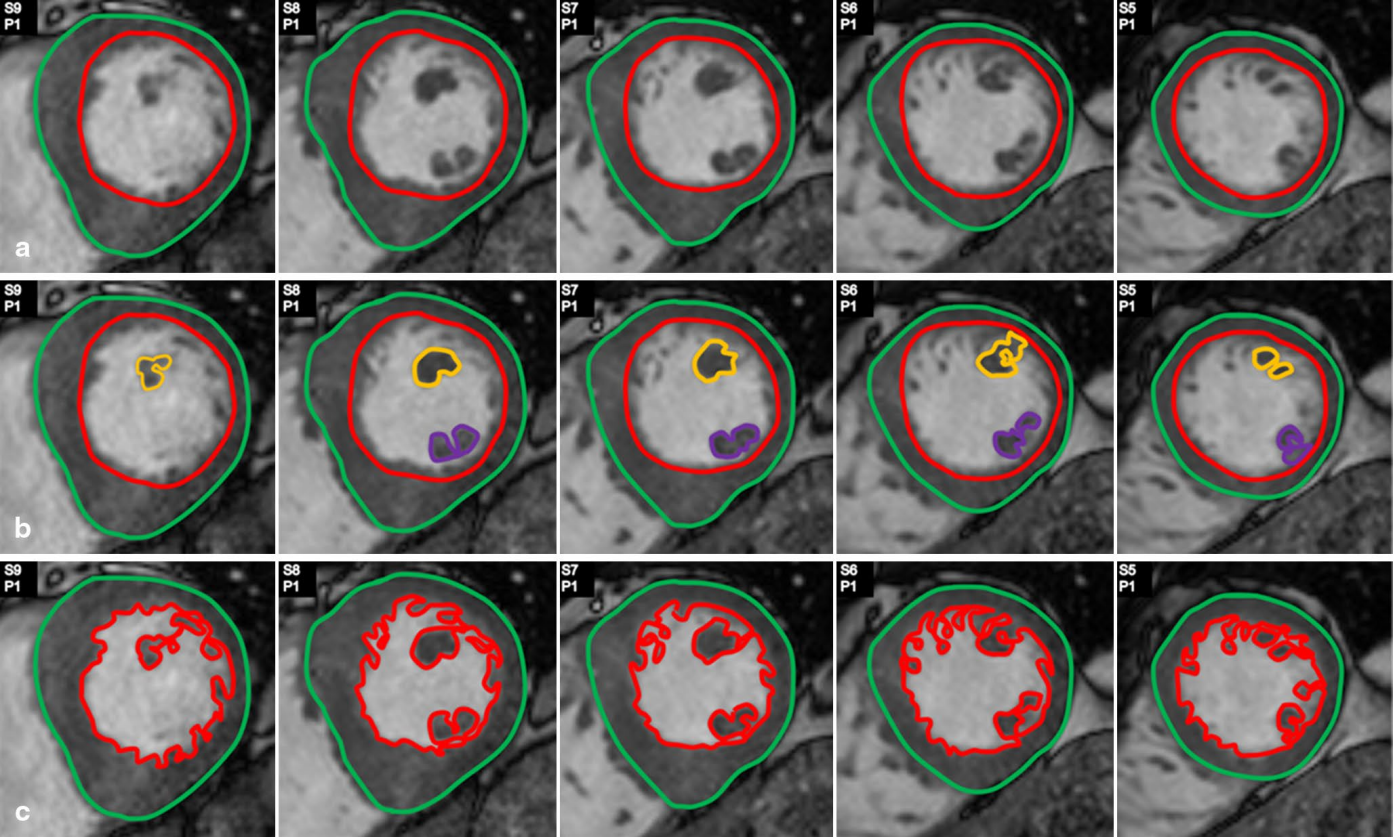
**Different methods of assessment of left ventricular (** *LV* **) parameters regarding the papillary muscles and trabecularisations.** Various image analysis protocols regarding the papillary muscles (PMs) and trabecularisations exist. In this study method A and B were compared. **a** LV mass and volume assessed with inclusion of the PMs in the LV blood pool. **b** LV mass and volume assessed with inclusion of the PMs in the LV mass. At the base of the PMs the circular shape of the endocardial contour was maintained to avoid inclusion of PM mass in the endocardial contour. **c** LV mass and volume assessed with inclusion of the PMs and the trabecularisations in the LV mass. Only those slices comprising papillary muscle are displayed

The endocardial and epicardial contours were manually drawn by one observer in the short-axis images in the LV end-diastolic and end-systolic phase using QMass® version 7.0 (Medis, Leiden, the Netherlands) (Fig. 1a). The end-diastolic phase corresponded to the first image in the cine sequence after ECG triggering. The end-systolic phase was chosen based on the smallest LV cavity at the mid-ventricular level. At the base of the heart, slices were included if more than 180° of the LV cavity was surrounded by LV myocardium [17].

Secondly, the PMs were defined on the short- and long-axis images as structures attached to the LV free wall and contiguous with the mitral valve via the chordae tendinae [14]. The contours of the PMs were manually drawn on the short-axis cine images for each slice that contained PM. A separate contour was used for the anterolateral and posteromedial PM. In case of accessory PMs, a continuous contour was used with two lines across the blood overlapping each other to avoid inclusion of LV blood pool as PM mass (Fig. 1b).

CMR imaging was performed using a 1.5 T MR scanner (Achieva, Philips Healthcare, Eindhoven, the Netherlands) with a cardiac coil. Cine images were acquired using a breath-hold ECG-triggered segmented SSFP sequence in 3 long-axis views (2-, 3-, and 4-chamber view) and in multiple short-axis views with a slice thickness of 10 mm (no gap), covering the LV from base to apex.

### LV parameters

The LVEF and LV mass were calculated as previously described [13, 18, 19]. These calculations were performed with results assessed according to two different protocols: (1) with inclusion of the PMs in the LV mass and (2) with exclusion of the PMs from the LV mass (Fig. 1a and b).

Absolute differences between results according to both protocols were calculated by subtraction. The absolute differences were transformed to relative differences for each LV parameter according to the following formula:

$$Relative\,difference\,in\,LV\,parameter=\frac{(result\,with\,inclusion\,of\,PMs\,in\,LV\,mass-result\,with\,exclusion\,of\,PMs\,from\,LV\,mass)}{Result\,with\,exclusion\,of\,PMs\,from\,LV\,mass}x100\%$$

PM mass was obtained according to the following formula:

$$PM\,mass=(LV\,mass\,with\,inclusion\,of\,PMs\,in\,LV\,mass)-(LV\,mass\,with\,exclusion\,of\,PMs\,from\,LV\,mass)$$

### Statistical analysis

LVEF, mass and PM mass were indexed to body surface area and expressed as mean ± SD. Within-group differences were compared using a paired samples t-test.

Between group differences were compared using an independent samples t-test. A p-value of < 0.05 was considered significant. Statistical analysis was performed with IBM SPSS Statistics 20.0 (IBM Corp, Armonk, NY, USA).

## Results

### Study population

**Fig. 2:**
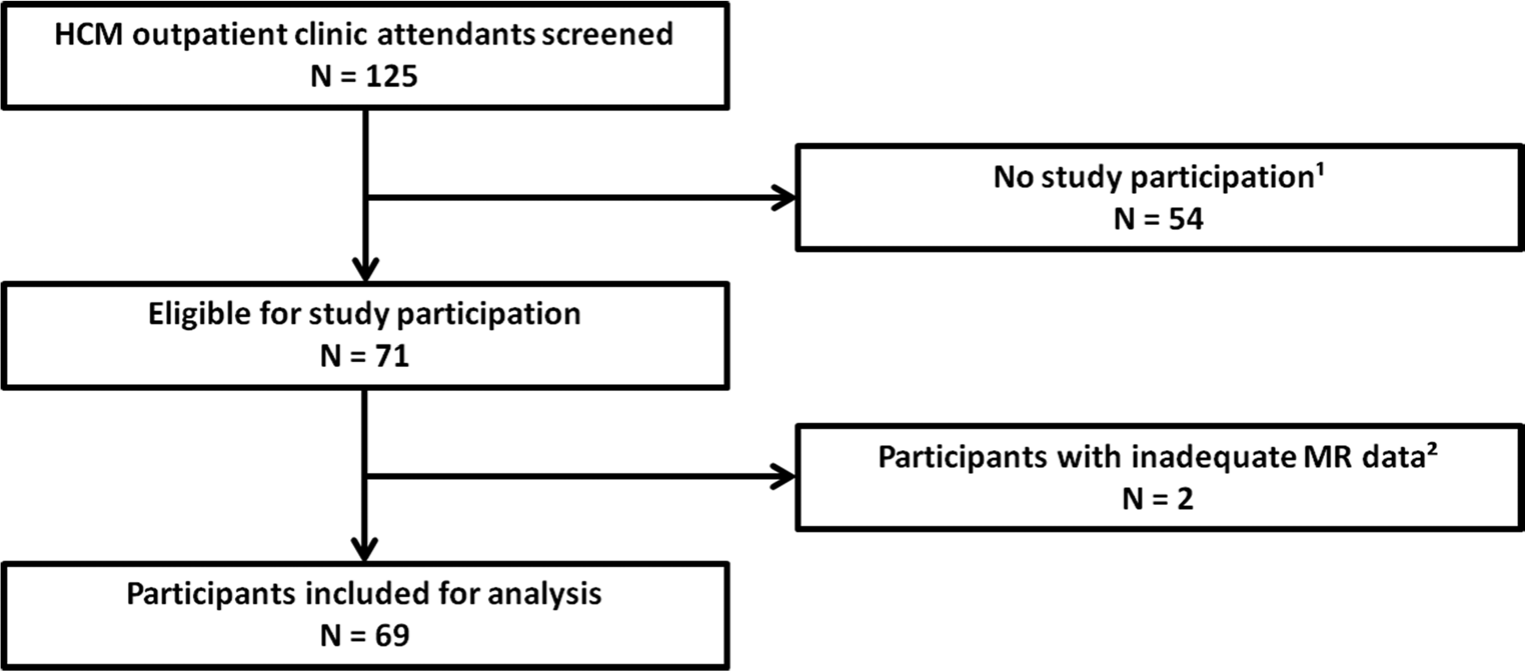
**Flowchart of study population.** *1*. Reasons for non-participation: 15 not willing to participate; 10 Morrow myectomy/Percutaneous transluminal septal myocardial ablation; 7 ICD or pacemaker; 5 could not be contacted; 5 claustrophobia; 12 other. *2*. Reasons for inadequate CMR imaging data: 1 claustrophobia; 1 inadequate CMR image quality

Of the 125 attendees of the outpatient clinic, 71 underwent CMR and in 2 data were inadequate for further analysis (Fig. 2). Finally, we analysed 69 subjects: 55 clinical HCM patients (with overt hypertrophy) and 14 subclinical HCM mutation carriers (without hypertrophy). Baseline characteristics are displayed in Table 1. Fifty-three clinical HCM patients (96 %) were asymptomatic to mildly symptomatic (NYHA 1–2) and most pathogenic mutations were located in the MYBPC3 gene (70 %).

**Table 1:** Baseline characteristics

	Total (*n* = 69)	Clinical HCM (*n* = 55)	Subclinical HCM mutation carriers (*n* = 14)
Age (years)	51 ± 16	54 ± 16	40 ± 10
Male	38 (55)	34 (62)	4 (29)
Pathogenic mutation present	37 (54)	23 (42)	14 (100)
NYHA class I/II	67 (97)	53 (96)	14 (100)
NYHA class III	2 (3)	2 (4)	–

Data are presented as mean ± standard deviation or numbers (percentages). *HCM* hypertrophic cardiomyopathy, *NYHA* New York Heart Association.

### Impact of the papillary muscles on LV parameters

The results for LVEF and LV mass are displayed per group in Table 2. For clinical HCM patients PM mass was 6.6 ± 2.5 g/m^2^. Inclusion or exclusion of the PMs resulted in relative differences of 4.5 ± 1.8 % for LVEF and 8.7 ± 2.6 % for LV mass (*p* < .01).

**Table 2:** Impact of inclusion or exclusion of the papillary muscles on LV ejection fraction and mass

**Clinical HCM (** *n* **= 55)**				
	*Exclusion of PMs from LV mass*	*Inclusion of PMs in LV mass*	*Absolute difference*	*Relative difference* ^a^
**LVEF (**%**)**	58.2 ± 7.2	60.8 ± 7.6	2.6 ± 1.1	4.5 ± 1.8
**LV mass (g/m** ^**2**^ **)**	78.5 ± 28.4	85.2 ± 30.3	6.6 ± 2.5	8.7 ± 2.6
**Subclinical HCM mutation carriers (** *n* = **14)**				
	*Exclusion of PMs from LV mass*	*Inclusion of PMs in LV mass*	*Absolute difference*	*Relative difference* ^a^
**LVEF (**%**)**	57.8 ± 6.1	60.0 ± 6.5	2.2 ± 0.7	3.8 ± 1.0
**LV mass (g/m** ^**2**^ **)**	42.7 ± 9.9	46.7 ± 10.5	4.0 ± 0.9	9.5 ± 1.8

Data are presented as mean ± standard deviation. Within group differences: *p* < .01.*LV* left ventricle, *EF* ejection fraction, *PMs* papillary muscles, *HCM* hypertrophic cardiomyopathy.^a^Relative differences in LVEF and mass are both indicated in percentages.

For subclinical HCM mutation carriers the PM mass was 4.0 ± 0.9 g/m^2^. The relative differences in LVEF and LV mass were 3.8 ± 1.0 % and 9.5 ± 1.8 % (*p* < .01) respectively, and not different from the relative figures observed in clinical HCM patients.

The observed absolute differences in LV mass differed significantly between groups 1 and 2 (*p* < .01), while the absolute differences in LVEF were not significant (*p* = 0.13).

## Discussion

To our knowledge this is the first study that addresses the basic question of how much inclusion or exclusion of the PMs affects everyday CMR image analysis in patients with HCM in particular, but also in relation to a group of controls without hypertrophy. In HCM patients, inclusion of the PMs resulted in significant relative increases of 9 % in LV mass and 4 % in LVEF. Despite the involvement of the PMs in the disease process of HCM, the relative differences were in the same order of magnitude in the control subjects without hypertrophic myocardium. This underscores the general need for uniform protocols, given the potential impact for clinical decision-making based upon values of LV mass, volume and function.

In recent studies on different CMR image analysis protocols, the PM mass was combined with that of the trabecularisations (Fig. 1c). Inclusion or exclusion of both the PMs and the trabecularisations as part of the LV mass led to significant differences in LVEF and LV mass [20–22]. This was confirmed in HCM patients [13, 23]. Reports on normal values with CMR imaging are limited and refer to studies that considered the PMs and trabecularisations to be part of the LV mass [10–12]. In daily practice, however, these structures are often not included, resulting in underestimation of LVEF and LV mass. Normal values for this more practical approach of CMR image analysis are scarce. Our study points out the need for either uniform CMR image analysis including these structures, or the need for studies on normal values of the more practical approach.

This is illustrated when our results are put in the context of the excellent reproducibility of CMR image analysis, with a coefficient of variation of 4 % [13, 17, 20, 24]. Based on the coefficient of variation we can make statistical inferences about the expected results when a particular LV mass is measured repeatedly, provided the same methodology of CMR image analysis is followed. Appreciating the 4 % coefficient of variation, the relative differences of the repeated measurements of that particular LV mass will lie between − 8 % and + 8 % in 95 % of cases. In a setting of uniform CMR analysis, this means that a true change in LV mass will be present if the results differ by more than a relative 8 %.

Our finding that the two different approaches of CMR analysis result in a 9 % relative difference in LV mass should be interpreted in this context. Given the mean relative difference of 9 %, the exact same LV mass will seem to have changed in at least half of the cases. This indicates that non-standardised assessment of LV mass may have important implications for both research and daily clinical practice. As for LVEF, the relative difference of 4 % seems less important.

In the setting of clinical trials, for example, there is a growing interest in studies reporting LV mass and the extent of fibrosis relative to LV mass [25, 26]. In case of CMR follow-up studies on this subject, CMR image analysis should be performed according to the same protocol at baseline and follow-up. Otherwise, differences in LV mass can be interpreted as a true change, while in fact it could be the result of two CMRs analysed according to two different protocols.

As for daily clinical practice, the use of CMR in HCM patients has increased over the years, even though it is not yet part of the routine clinical work-up for risk stratification [1]. This development is partly related to its excellent reproducibility, but may also be influenced by the growing body of evidence reported by CMR studies in the field of HCM. For example, increased LV mass assessed by CMR (i.e. > 91 or > 69 g/m^2^ for men and women respectively) has been suggested as a more sensitive marker of adverse outcome than LV maximal wall thickness [6]. A cut-off value (< 50 %) has been reported to predict an adverse prognosis in HCM, not only for LV mass but also for LVEF [4, 5]. In the latter case, the 4 % difference we observed could have implications for patients with values near the cut-off. In that regard, it has previously been demonstrated that depending on the imaging technique, differences in LVEF assessment may have important clinical consequences in ICD candidates [27]. In addition, the amount of fibrosis relative to LV mass has the potential to become an additional risk factor in the selection of candidates for primary prevention with an ICD [2, 3]. Again, this underscores the importance of uniform assessment of LV mass.

In order to achieve uniformity, a guideline statement with regard to the preferred method of analysis should be a first initiative. In the above-mentioned context of increasing use of CMR data, the recent initiative to validate the accuracy of SSFP CMR imaging should be appreciated [28]. Importantly, these figures on accuracy were obtained with inclusion of both the PMs and the trabecularisations. On the other hand, algorithms incorporating quantification of trabecularisations have been questioned with regard to reproducibility, given the possible overestimation of LV mass due to partial volume artefacts [17, 29]. If exclusion of the PMs were to become the method of choice, normal values obtained with contemporary sequences in an adequately sized series of healthy controls would be required [30]. In the present era of non-uniform CMR analysis, reliable comparison between previous and future studies is difficult. This underscores the need for investigators to report the CMR analysis algorithm, and clinicians should be aware of the protocol used at their centre when interpreting CMR parameters.

In the present comparison of the two algorithms, the associated absolute differences in LV mass were higher in clinical HCM patients than in subjects who were HCM mutation carriers without hypertrophy. On the other hand, the relative changes in LVEF and LV mass were similar in both groups. This can be explained by the fact that in HCM patients the proportion of the PM mass in relation to the total LV mass was similar to that in subjects without hypertrophy. Subsequently, after transformation of the absolute differences to relative differences, the impact of inclusion or exclusion of the PMs was comparable in both groups. This suggests that our results apply not only to patients with overt hypertrophy, but may apply to the general population as well, although more study subjects are necessary to substantiate this. This is supported by a study in healthy controls, which reported a similar impact of the PMs on LV mass [31].

It should be appreciated that this study did not address accuracy, due to lack of a comparative standard. We merely described the impact of a more practical CMR algorithm in terms of quantification of LV parameters, and with regard to potential consequences for research questions and daily clinical practice. In order to put our findings into context, we referred to the well-accepted 4 % coefficient of variation, i.e. a relative difference of 8 % or more indicates a true change [13, 17, 20, 24]. In a random set of 20 of our participants, intraobserver and interobserver variability were 4.69 and 4.49 % for LVEF and 4.76 and 4.86 % for LV mass, respectively.

In summary, our findings indicate that non-uniform CMR image analysis will render incorrect conclusions with regard to the presence or absence of changes in important LV parameters in about half of patients with two CMR assessments. This holds true not only for HCM patients with overt hypertrophy but also for subjects without hypertrophy. Given the potential impact for both research and daily clinical practice, our data underscore the importance of a standardised approach, either with or without the PMs as part of the LV mass.
